# Kiwi Root Extract Inhibits the Development of Endometriosis in Mice by Downregulating Inflammatory Factors

**DOI:** 10.1155/2021/4536132

**Published:** 2021-01-26

**Authors:** Tingting Liao, Shenzhi Zhao, Tong Zhou, Jiajia Song, Xianping Huang, Huiqiu Xiang, Yanyan Lin, Jiajia Chen, Zhangye Xu

**Affiliations:** ^1^Department of Gynecology and Obstetrics, The Second Affiliated Hospital of Wenzhou Medical University, Wenzhou 325027, China; ^2^Wenzhou Medical University, Wenzhou 325027, China

## Abstract

**Purpose:**

To determine whether the kiwi root extract inhibits the development of endometriosis in mice by suppressing inflammatory factors.

**Materials and Methods:**

The mouse model of endometriosis was induced by surgery after which the mice were continuously injected with the drug for 14 days. On the 14^th^ day, the mice were sacrificed, and the peritoneal fluid was obtained for enzyme-linked immunosorbent assay. Endometrial ectopic tissue was weighed and analyzed by tissue immunochemistry, RT-PCR, western blotting, and gelatin zymography experiment.

**Results:**

Kiwi root extract significantly reduced endometriotic lesion volume and downregulated the proinflammatory cytokines IL-6, IL-8, IL-1*β*, and TNF-*α*, as well as the angiogenic factor VEGF-A. It also inhibited the mRNA and protein expression of COX-1 and COX-2, IL-6, TGF-*β*1, EP_2_ receptor, and ER-*β* in endometriotic lesions but did not affect the expression of MMP-9 and MMP-2.

**Conclusions:**

Kiwi root extract could significantly inhibit the growth of surgery-induced endometriosis in mice. Our results suggest that the kiwi root extract may inhibit the development and progression of ectopic endometrium through disruption of neovascularization and reducing inflammation, which may be beneficial in treating this common gynecological disease.

## 1. Introduction

Endometriosis is a chronic, inflammatory, hormone-dependent gynecological disease characterized by the growth of the extrauterine matrix and endometrial glands [1]. It causes chronic pelvic pain, dysmenorrhea, difficulty in sexual intercourse, premature birth, infertility, and similar [2]. There has not been a standard treatment for endometriosis. Current treatments have been associated with adverse side effects and recurrence of the disease after discontinuation of therapy.

The progression of endometriosis is characterized by marked alternations in estrogen metabolism and proinflammatory mediator levels and activity [3, 4]. The growth of endometrial cells implies “adhesion invade-angiogenesis”, similar to the biological behavior of malignant tumor cells. Increased activation of macrophages in peritoneal fluid in patients with endometriosis leads to the upregulation of proinflammatory factors, such as IL-1*β*, IL-6, IL-8, and TNF-*α*, which create favorable conditions for their occurrence [5, 6]. TNF-*α* is the most important inflammatory mediator in the process of an inflammatory reaction, which can induce the release of ectopic endometrial inflammatory cytokines MCP-1, IL-6, and IL-8. Simultaneously, it is also beneficial for ectopic endometrium and stromal cell proliferation of inflammatory cells since it facilitates their infiltration [7]. Estrogen mediates the mesenchymal transition of endometrial epithelial cells [8, 9]. Han et al. [9] found that the endometriotic steroid receptor coactivators may prevent TNF-*α* mediated apoptosis in human endometrial epithelial cells and cause the epithelial-mesenchymal transition and the invasion of human endometrial cells, which are considered as hallmarks of advanced endometriosis. At endometriotic sites, inflammatory cells can generate reactive oxygen species that contribute to oxidative stress in the peritoneal cavity. Oxidative stress further augments immune response in affected sites [10, 11].

Chinese kiwi is considered a herbal medicine with therapeutic value. Its flesh, peel, and root can be used to treat various diseases [12]. Previous studies have found that *Actinidia chinensis* root extract has anti-inflammatory effects and participates in inhibiting tumor growth [13–15]. The aim of this study was to examine the effect of the kiwi root extract and its underlying mechanisms in the treatment of endometriosis, mainly focusing on inflammatory and angiogenesis factors as well as the involvement of estrogen receptors.

## 2. Materials and Methods

### 2.1. Dissolution of the Kiwi Root Extract

Kiwi root extract was purchased from Zi Xing Mu Fang Company. The main component of the extracted powder is triterpenes (such as coconut acid); other compounds included 2*β*, 3*β*, 23-trihydroxy-urs-12-ene-28-oleic acid and polysaccharides. Fresh kiwi roots were processed using several pressures (extraction by water, concentration, dry, crushing, and sieving) to obtain the pure kiwi root extract. Next, 3 g kiwi root extract powder was dissolved in 38 ml double-distilled water. Finally, a concentration of 78.9 mg/ml liquid drug was obtained after sterilization with high pressure.

### 2.2. Animals

BALB/c mice (6 weeks old; 16–18 g in weight) were purchased from Beijing Weitong Lihua. Mice were maintained under pathogen-free conditions in a controlled environment with temperatures between 20°C and 25°C and 12-hour cycles of light and dark. After 2 weeks of acclimatization, mice were used for surgical modeling (8 weeks of age). All animal studies were done in compliance with the regulation and guidelines of Wenzhou Medical University institutional animal care and conducted according to the AAALAC and the IACUC guidelines.

#### 2.2.1. Surgical Induction of Endometriosis

The surgical method used to induce endometriosis is based on our previous research and the research of other scholars [16]. Endometriosis was induced using homologous uterine horn transplantation. Briefly, the body hair on the lower abdomen's left and right sides was removed, and the abdomen was disinfected with alcohol cotton. Clean surgical scissors were used to cut a small opening on the left and right abdomen without touching the peritoneum. The surgical tweezers, which were held in the right hand, were used to clamp a small section of the donor uterine fragments in the dish into the incision, buried in the inner abdominal wall and the outer peritoneum, without touching the peritoneal cavity. Smooth bulging endometriotic lesions were observed in all the experimental animals after two weeks. A developed mouse model of endometriosis revealed histopathological components (glands and stroma) typical of endometriotic lesions.

#### 2.2.2. Preliminary Experiment to Determine the Concentration of the Treated Group

The donor mouse uterine fragments were transplanted into the abdominal wall of the recipient mice. The mice after surgery were randomly divided into five groups (6 per group): normal saline group and four experimental groups (administered with 300, 600, 900, and 1200 mg/kg/d, respectively). The daily injection volume was 300 ul per day. Each group of mice was weighed before the first injection and then after every 3 days. After two weeks, five groups of mice were sacrificed by cervical dislocation. Furthermore, abdominal fluid was obtained for ELISA detection after centrifugation. According to the experimental results, the dose of the treated group was determined to be the dose of the group with the most significant decrease in inflammation indicators.

#### 2.2.3. Formally Studied Specimen Collection

Endometriosis mice were randomly divided into two groups (6 per group): the treated group that received 600 mg/kg/d dose of kiwi root extract solution by intraperitoneal injection on a daily basis (300 ul) for 2 weeks, and the control group that received normal saline (300 ul). On the day of the last injection, the mice were sacrificed and endometriotic lesion tissues were identiﬁed by pathology. Then, we calculated the transplantation rate. One investigator who was blinded to the groups' researcher measured the size and the relative volume of endometriotic lesions using the same calipers as the ﬁrst researcher. Lesions were flash-frozen in liquid nitrogen for RT-PCR, fixed in 10% neutral buffered formalin for 24 hours, and embedded in paraffin for immunohistochemistry. Then, 1 ml of sterile PBS was injected into the abdominal cavity, gently massaging for collecting the peritoneal fluid better. Peritoneal lavage supernatants were transferred to sterile Eppendorf and stored at −80°C.

### 2.3. Real-Time Quantitative RT-PCR

Total RNA was extracted following the TRIzol reagent kit instructions (Invitrogen, USA). Reverse transcription was performed according to Takara kit manufacturer's instructions to prepare corresponding cDNA which was amplified after reverse transcription; the conditions of amplification reaction were 95°C, predenaturation for 1 cycle, 95°C, 10 s, 52°C, 10 s, and 72°C, 10 s for a total of 45 cycles to determine the corresponding dissolution curve. Relative quantitative analysis of mRNA *F* = 2 −∆∆Ct, and standard PCR amplification efficiency were determined. The primer sequences are shown in Table 1.

### 2.4. Western Blot

Total protein was extracted from the specimen with TRIzol. BCA Protein Assay Kit (Beyotime, Haimen, China) was used to detect the protein concentrations. 50 *μ*g protein samples were resolved on 12% SDS polyacrylamide gels and transferred to polyvinylidene fluoride membranes. After being blocked with 5% free-fat milk in Tween 20-containing Tris-buffered saline for 2 hours, the membranes were washed with TBST and separately incubated with antibodies against COX-1, COX-2 (1 : 1000; Abcam, Biotechnology), *β*-actin, IL-1*β*, mPGE_s_ (1 : 1,000; Affinity, USA), IL-6, and ER-*β* (1 : 1000; Abcam, Biotechnology) at 4°C overnight. The next day, after washing in TBST, membranes were incubated with secondary antibodies (1 : 3000 dilution Bioword, MN, USA) for 1.5 h at room temperature. Then, membranes were rewashed in TBST, and the protein bands were visualized with enhanced chemiluminescence. The intensities of proteins were quantified using Image *J* software.

### 2.5. Immunohistochemical Staining

A 5 *μ*m tissue section was cut from paraffin-embedded blocks and adhered to and dried on poly-L-lysine precoated glass slides. Sections were deparaffinized and rehydrated after 1.5 h incubation at 65°C, after which they were treated for ten minutes with xylene in order to remove the paraffin. The sections were rehydrated using 100%, 95%, 85%, 75% alcohol, and water (5 min for each step). Consequently, sections were washed with 1X PBS (130 mM NaCl, 7 mM Na2HPO4, 3 mM KH2PO4, pH 7.4) and incubated for 2 hours at room temperature with bloc 0.1% Triton X-100 (GENE Ray). Endogenous peroxidase activity was inhibited by incubation for 10 min with 0.3% hydrogen peroxide, after which the slides were heated for 15 minutes in 10 mmol/L citrate buffer (pH 6.0) in a microwave oven to achieve antigen retrieval. Goat serum was added to slides to prevent nonspecific binding. Slides were transferred in primary antibodies ER-*β* (1 : 100), VEGF-A (1 : 100), PGE_2_ (1 : 100), EP_2_ (1 : 100), COX-1 (1 : 100), and COX-2 (1 : 100) overnight at 4^o^C. The next day, after 45 minutes of rewarming at 37 degrees, slides were washed three times with PBS (0.1% Triton X in 1X PBS) for 2 minutes each. After washing, goat anti-rabbit antibody was used as the secondary antibody, and microscopically controlled chromogenic DAB dyeing nuclei were stained with hematoxylin. Positive staining was indicated by the yellow or brown color of the cytoplasm or nucleus. The cumulative light density value of the images was obtained and calculated by using Image-Pro Plus (IPP) software.

### 2.6. Enzyme-Linked Immunosorbent Assay (ELISA)

Ascites were collected into collection tubes, centrifuged at 2,000 rpm for 10 minutes, and the supernatants were immediately stored at −80°C until use. The samples were treated with an enzyme-linked immunosorbent assay (ELISA) kit according to the manufacturer's instruction (Chemicon, CA, USA). Briefly, assay diluent and samples were added to precoated wells of 96-well plates. The plates were incubated at room temperature for several hours and then washed five times with washing buffer. The plates were incubated with specified secondary antibody conjugated with peroxidase at room temperature for 2 hours, after which peroxidase was introduced for 30 minutes followed by the stop solution. Absorbance was read at 450 nm. GraphPad Prism 5 software was used to organize data analysis. In the preliminary experiment, we tested the three factors of IL-1*β*, COX-1, and PGE_2_. Furthermore, we detected the three factors of TNF-*α*, TGF-*β*1, and VEGF-A in the formal experiment.

### 2.7. Gelatin Zymogram

Gelatin zymography assay activity of MMP-2 and MMP-9 was assessed in the peritoneal fluid by gelatin zymography. Proteins of endometriosis tissue were collected, and the protein concentration of each group was adjusted by mixing with a 5x loading buffer. After SDS-PAGE electrophoresis at 4°C with 100 v for about 1.5 hours, the gel was placed in the eluent and shaken twice for several times. Then, the gel was placed in the incubation solution and incubated at 37 C for 42 hours, after which it was stained for 3 hours and, respectively, decolorized for 0.5, 1, and 2 hours in the decolorizing liquids A, B, and C, showing the translucent bands of MMP-2 and MMP-9.

### 2.8. Statistical Analysis

Statistical analysis was performed using SPSS 17.0 statistical software. All data are expressed as mean ± SEM and *P* values with a *t*-test. A *P* value <0.05 was considered statistically significant.

## 3. Results

### 3.1. Preliminary Experiment Selection of  the Dose of the Treated Group

**Figure 1:**
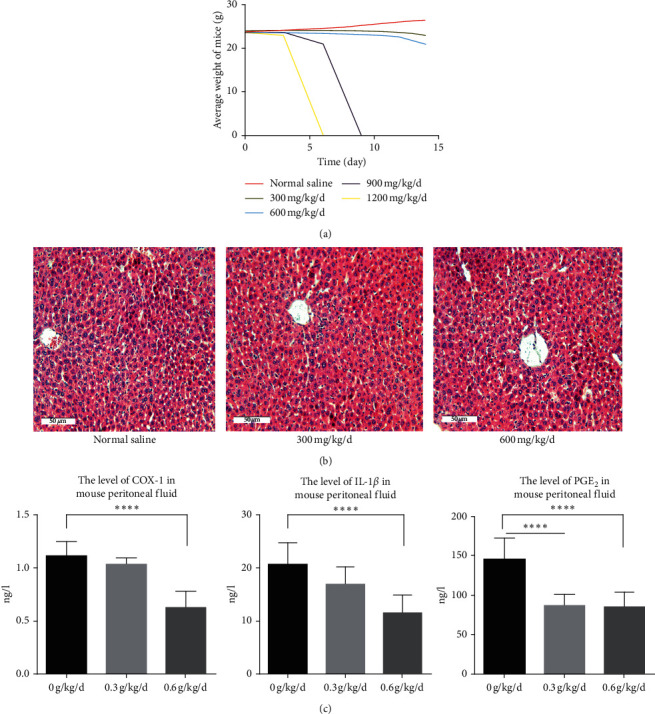
The changes in indicators of each group of mice after treatment with the kiwi root extract. (a) The picture of the average body weight of each group of animals after drug injection over time. (b) Representative liver hematoxylin-eosin staining images of surviving mice. (c) The relative expression level of COX-1, IL-1*β*, and PGE_2_ protein in 0 g/kg/d, 0.3 g/kg/d, and 0.6 g/kg/d groups of mice was detected by ELISA.

The preliminary experiment results show that no significant changes were found in mice treated with different drug concentrations during the first 3 days of treatment. After injection of the drug, the urine color was darker, and fecal output increased. The weight of the mice treated with the drug concentration of 900 and 1200 mg/kg/d gradually decreased over time. All mice treated with 1200 mg/kg/d and 900 mg/kg/d died after 6 and 9 days, respectively. All mice in the remaining groups survived but showed gradual weight loss (Figure 1(a)). The mice that survived after 2 weeks were sacrificed by cervical dislocation. The peritoneal fluid was obtained and the serum samples were analyzed using ELISA [17]. No hepatotoxicity was found after treatment (Figure 1(b)). Considering that the proinflammatory factors of the 600 mg/kg/d group were significantly lower than those of the control group, 600 mg/kg/d was selected as the dose of the treated group (Figure 1(c)).

### 3.2. Kiwi Root Extract Inhibits the Growth of Endometriotic Tissue

**Figure 2:**
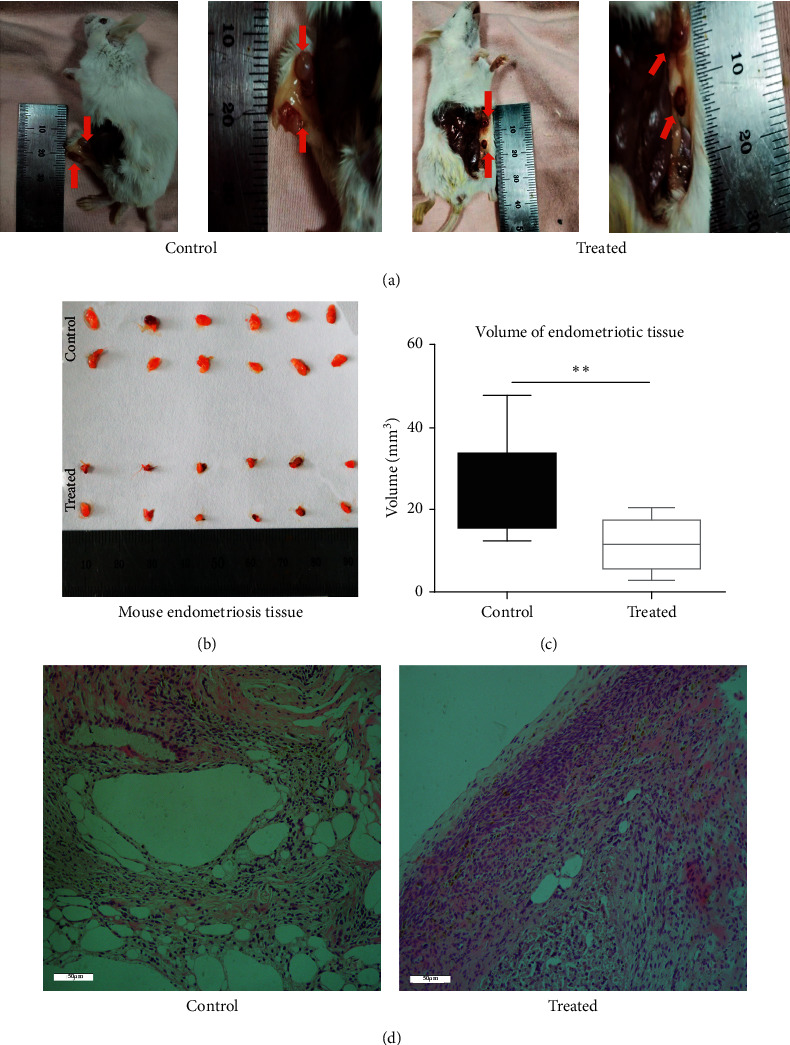
Treatment with the kiwi root extract reduces the growth of endometriosis. (a) Picture of representative mice in the control group and the treated group. (b) Endometriosis tissues of the control group (control) and treated group (treated) were taken out of mice. (c) Endometriotic tissue volume, as shown in the picture; the endometriosis tissue volume of the control group (24.44 ± 3.219) is higher than the endometriosis tissue volume of the treated group (11.66 ± 1.813) (*P* = 0.002). (d) Representative hematoxylin-eosin staining images of the mouse endometriosis control group and treated group taken with 20× objective lens.

Female BALB/c recipient mice were administered with 600 mg/kg/d of a drug for 14 days after surgery. Then, the uterus's ectopic tissue was dissected and analyzed. Mice in both groups (treated and control groups) showed an evident cyst-like lesion on the peritoneal wall, which adhered to the inside of the abdominal wall or lateral peritoneum; yet, a more significant reduction in lesion volume was observed in the treated mice compared to the control mice (Figures 2(a)–2(c)), while the amount of transplanted endometriotic tissue in the mice was not affected. A histopathological examination showed that the ectopic endometrial tissue had one or more cavity-like structures, including luminal surface epithelium, stromal cell membrane, and lamina propria. However, treatment with the kiwi root extract resulted in a more rudimentary architecture with less developed glands surrounded by little stroma (Figure 2(d)).

### 3.3. Kiwi Root Extract Downregulates Inﬂammation-Associated Factors in Endometriosis

**Figure 3:**
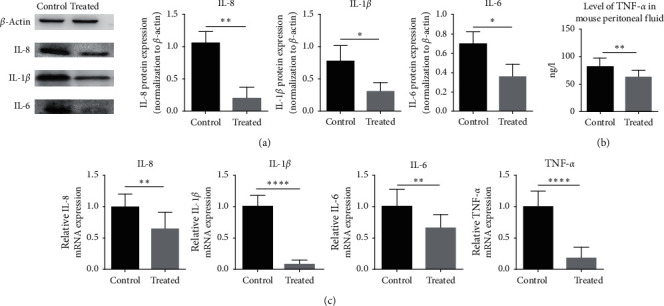
The expression of IL-6, IL-8, IL-1*β*, and TNF-*α* mRNA and protein in the endometriotic tissue of different groups. (a) The relative expression level of IL-6, IL-8, and IL-1*β* protein was detected by western blot. (b) The relative expression level of TNF-*α* protein was detected by ELISA. (c) The relative expression level of IL-6, IL-8, IL-1*β*, and TNF-*α* mRNA was detected by RT-PCR. Data are expressed as mean ± standard deviation from triplicate experiments (^*∗*^*P* < 0.05, ^*∗∗*^*P* < 0.01, ^*∗∗∗*^*P* < 0.001, and ^*∗∗∗∗*^*P* < 0.0001).

Next, RT-PCR and western blot were used to assess changes in inflammation-associated factors in endometriosis. From a genetic perspective, the results showed that the expression of IL-6, IL-8, IL-1*β*, and TNF-*α* mRNA in the control group (1.000 ± 0.0, *N* = 6) significantly increased compared to that in the treated group (0.6570 ± 0.07292, *N* = 6; 0.6528 ± 0.08644, *N* = 6; 0.08509 ± 0.02157, *N* = 6; 0.1759 ± 0.05792, *N* = 6, respectively, Figure 3(c)). In addition, the levels of IL-6, IL-8, and IL-1*β* protein were markedly downregulated in the treated group compared to those in the control group (Figure 3(a)). Meanwhile, ELISA assays demonstrated that there was a trend of decreasing TNF-*α* protein in the Treated group (62.25 ± 3.425 *N* = 6) compared to the Control group (81.36 ± 4.551 *N* = 6) (Figure 3(b)).

### 3.4. Kiwi Root Extract Blocks the Vascular Endothelial Growth Factor (VEGF-A)

**Figure 4:**
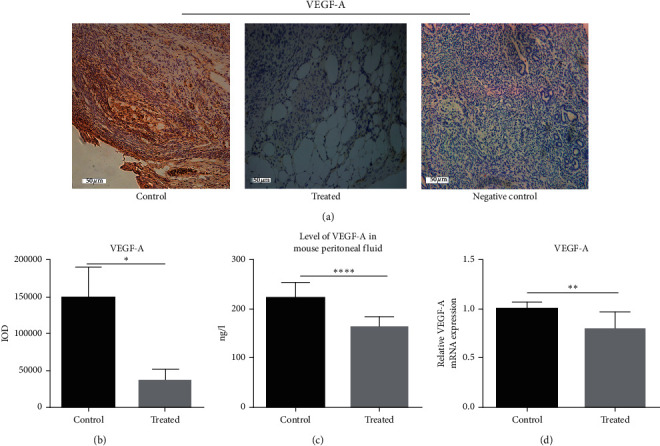
The expression of VEGF-A mRNA and protein in the endometriotic tissue of different groups. (a) Representative immunohistochemical images of mouse endometriosis control group and treated group taken with 20× objective lens (×200). The scale bar represents 50 microns. (b) The relative expression level of VEGF-A protein was detected by immunohistochemical staining. (c) The relative expression level of VEGF-A protein was detected by ELISA. Data are expressed as mean ± standard deviation from triplicate experiments. (d) The relative expression level of VEGF-A mRNA was detected by RT-PCR (^*∗*^*P* < 0.05, ^*∗∗*^*P* < 0.01, ^*∗∗∗*^*P* < 0.001, and ^*∗∗∗∗*^*P* < 0.0001).

After treatment, RT-PCR revealed that the expression of VEGF-A mRNA (1.000 ± 0.0) was significantly higher in the endometriotic tissue of the control group than that of the treated group (0.7945 ± 0.05580; Figure 4(d)). This was further confirmed by immunohistochemistry (Figure 4(a)), and the difference was statistically significant (*P* < 0.05) (Figure 4(b)). As expected, ELISA of peritoneal fluid samples showed a downward trend of VEGF-A (Figure 4(c)), where the expression of VEGF-A protein was appreciably reduced in the Treated group (37082 ± 8426) compared with the Control group (148941 ± 23611).

### 3.5. Kiwi Root Extract Modulates the Expression of TGF-*β*1 in Endometriotic Lesions but Does Not Affect the Expression of MMP2 and MMP9

**Figure 5:**
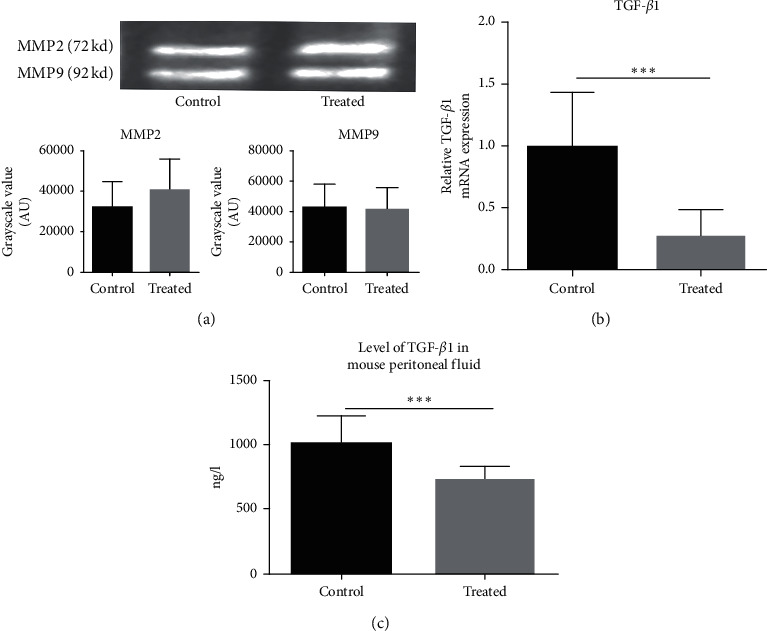
The expression of TGF-*β*1, MMP2, and MMP9 mRNA and protein in endometriotic tissue of different groups. (a) The relative expression level of MMP2 and MMP9 protein was detected by gelatin zymography. Data are expressed as mean ± standard deviation from triplicate experiments. (b) The relative expression level of TGF-*β*1 mRNA was detected by RT-PCR. (c) The relative expression level of TGF-*β*1 protein was detected by ELISA (^*∗*^*P* < 0.05, ^*∗∗*^*P* < 0.01, ^*∗∗∗*^*P* < 0.001, and ^*∗∗∗∗*^*P* < 0.0001).

The gelatin zymography results showed that the Treated group was not significantly different from the Control group in the expression of MMP2 and MMP9 (*P* > 0.05) (Figure 5(a)). Yet, the kiwi root extract dramatically decreased the expression of TGF-*β*1 mRNA and protein in the treated group compared to the control group (*P* < 0.001) (Figures 5(b) and 5(c)).

### 3.6. Kiwi Root Extract Reduces the Expression of COX-1, COX-2, EP_2_, PGE_2_, and mPGEs

**Figure 6:**
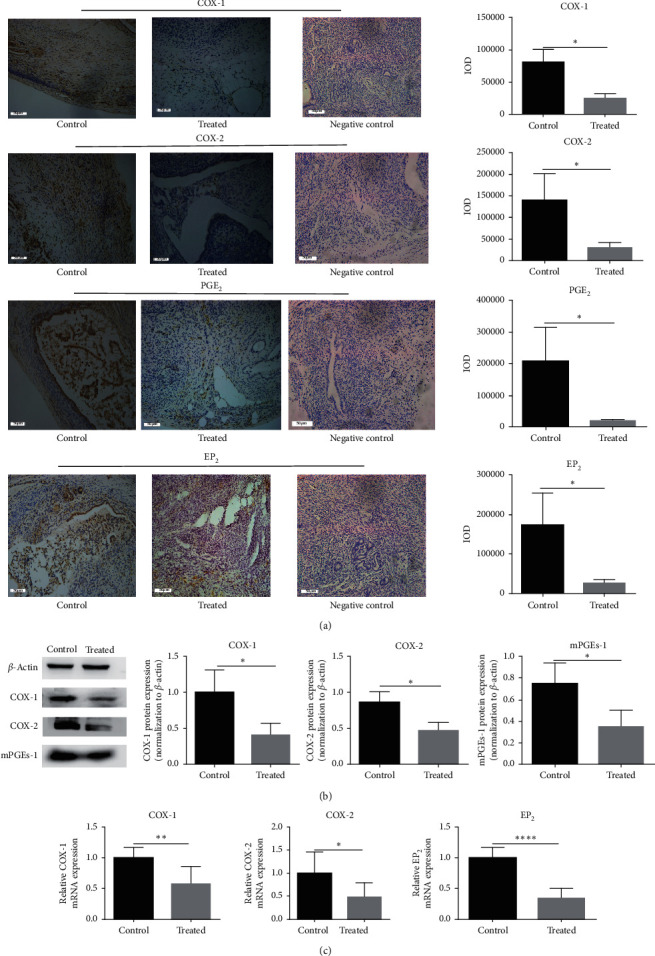
The expression of COX-1, COX-2, and EP2 mRNA and the expression of COX-1, COX-2, mPGEs, EP_2_, and PGE_2_ protein in endometriotic tissue of different groups. (a) The relative expression level of COX-1, COX-2, PGE_2_, and EP_2_ protein was detected by immunohistochemical staining (×200); the scale bar represents 50 microns. (b) The relative expression level of COX-1, COX-2, and mPGEs protein was detected by western blot. Data are expressed as mean ± standard deviation from triplicate experiments. (c) The relative expression level of COX-1, COX-2, and EP2 mRNA was detected by RT-PCR (^*∗*^*P* < 0.05, ^*∗∗*^*P* < 0.01, ^*∗∗∗*^*P* < 0.001, and ^*∗∗∗∗*^*P* < 0.0001).

As shown in Figure 6(a), COX-1, COX-2, PGE_2_, and EP_2_ protein expression were reduced in the endometriotic tissue of mice treated by kiwi root extract (*P* < 0.05). The expression of COX-1, COX-2, and mPGEs by western blot results also showed the downtrend in the treated group (*P* < 0.05) (Figure 6(b)). Moreover, the mRNA expression levels of COX-1, COX-2, and EP_2_ in the treated group were lower than those in the control group (*P* < 0.05) (Figure 6(c)).

### 3.7. Kiwi Root Extract Inhibits Estrogen-Regulated Genes in Endometriotic Lesions

**Figure 7:**
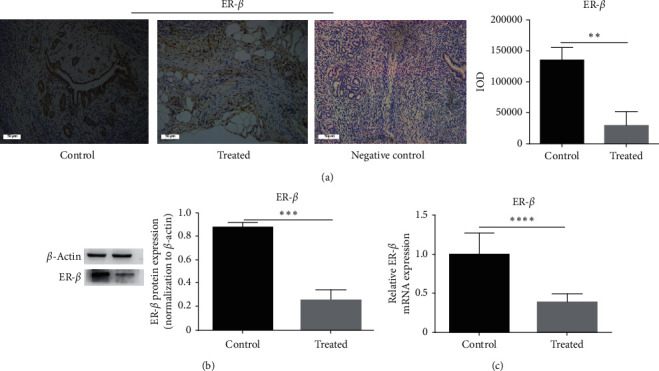
The expression of ER-*β* mRNA and protein in the endometriotic tissue of different groups. (a) The relative expression level of ER-*β* protein was detected by immunohistochemical staining (×200); the scale bar represents 50 microns. (b) The relative expression level of ER-*β* protein was detected by western blot. Data are expressed as mean ± standard deviation from triplicate experiments. (c) The relative expression level of ER-*β* mRNA was detected by RT-PCR.

As shown in Figure 7(c), kiwi root extract affected endometriotic lesions by changing the level of estrogen-regulated genes such as ER-*β* (*P* < 0.05). The expression of ER-*β* protein was violently altered after kiwi root treatment (Figures 7(a) and 7(b)). Estrogen can promote the proliferation of endometrial tissue by activating specific genes involved in the cell cycle [18]. Therefore, the impact on estrogen receptors will inevitably have a certain impact on the occurrence and development of endometriotic tissues.

## 4. Discussion

Kiwi root extract is considered a natural Chinese herbal medicine that possesses anti-inflammatory and antitumor proliferation properties [19]. Triterpenoids and phenolic acids, which are one of the main extracts of kiwi root, have shown to possess significant antitumor activity [20]. Moreover, recent studies have also shown that kiwi root has an inhibitory effect on various tumors [15, 21]. Endometriosis is a chronic, inflammatory, hormone-dependent gynecological disease with a biological behavior that is similar to malignant tumor cells [22]. In the present study, we evaluated the effect of kiwi root extract on endometriotic lesions in mice.

Kiwi root extract caused a significant inhibition of endometrial growth, including the shrinkage of endometriotic lesions and the atrophy of endometrial epithelium and glands. Endometrial cells undergo adhesion, growth, and progression, which may eventually lead to endometriosis. Inflammation and oxidative stress during this process may be associated with the development of endometriosis [22]. Oxidative stress produces inflammatory cytokines, including IL-6, IL-8, IL-1*β*, and TNF-*α*. In turn, these proinflammatory cytokines induce oxidative stress. Consequently, the vicious cycle between oxidative stress and inflammatory cytokines commences when the process becomes chronic. Therefore, in this study, we first tested a panel of inflammation-associated factors, including IL-6, IL-8, IL-1*β*, and TNF-*α*. Briefly, kiwi root extract profile may be related to endometriosis.

VEGF-A is an essential factor involved in the pathogenesis of endometriosis. It activates intracellular pathways and related signaling molecules that promote angiogenesis. The inhibition of VEGF-A may be a potential therapeutic target for the treatment of endometriosis [23, 24]. IL-1*β* and TGF-*β*1, which can regulate the expression of VEGF-A, participate in the formation of new blood vessels and promote the metastatic spread of tissues [25]. In addition, MMPS and TGF-*β*1 are involved in the degradation of extracellular matrices that are commonly observed in endometriosis development. In the present study, we examined MMP2 and MMP9 by gelatin zymography. The results suggested that there is no significant difference in MMP2 and MMP9 between the control group and the treated group. Yet, VEGF-A and TGF-*β*1 in the drug-administered group were significantly lower than those in the control group. These results suggested that kiwi root extract prevents neovascularization in endometriosis by downregulating VEGF-A through TGF-*β*1and IL-1*β* and not through the MMPS family.

Cyclooxygenase-1 (COX-1) and COX-2 are inducible enzymes that mediate prostaglandin synthesis, which has a vital role in the inflammatory response [26]. The COX-2 of ectopic endometrial tissue is higher, which increases the number of enzymes produced by COX-2 in ectopic tissues and upregulates the expression of proinflammatory factor PGE_2_ [27]. PGE_2_, in turn, strongly induces CYP19a1 (aromatase), thus creating a positive feedback mechanism that maintains local estradiol levels [28, 29]. In this study, we found that kiwi root extract reduces prostaglandin E2 synthetase, thus resulting in a decrease of prostaglandin E2. It has been reported that the inflammatory environment of ectopic tissue is the first step in the development of ectopic tissue and that its upstream factor IL-1*β* [30] can control the expression of COX-2. Moreover, COX-2 and its metabolites have a crucial role in inflammation-tumor transformation [31]. Our study prompts that the kiwi root extract may reduce the COX-2 expression by downregulating IL-1*β*, which is a key inflammatory factor, resulting in a decrease of mPGEs and PGE_2_. In addition, estrogen production and signaling are considered central to the pathogenesis of endometriosis [4, 29]. Accordingly, in our surgically induced model, we observed decreased ER-*β* and COX-2 expression in the endometriotic lesions of treated mice. Downregulation of ER-*β* expression disrupts pyrophosphorylation and apoptotic signaling in endometriotic lesions [32]. Regulation of ER-*β* affects cell proliferation and early apoptosis. Furthermore, it may have an important role in the growth of embryonic stem cells [33].

In conclusion, these data indicated that the kiwi root extract might inhibit endometriosis progression through multiple local mechanisms. Kiwi root extract regulates mediators responsible for diverse biological activities, such as inflammation (IL-1*β*, IL-6, and COX-2), cell adhesion, and invasion (TGF-*β*1, VEGF-A). It also directly affects epithelial and stromal cells and exerts its action by downregulating inflammation factor and vascular endothelial factor, thus disrupting neovascularization and the microenvironment of endometriosis growth. Thus, kiwi root extract might be a potential drug candidate for the treatment of endometriosis.

## Figures and Tables

**Table 1:** Oligonucleotide sequences used for RT-PCR.

Target mRNA	Primer	Sequence	PCR product (bp)
GAPDH	Forward	5′-TGACTTCAACAGCGACACCCA-3′	121
	Reverse	5′-CACCCTGTTGCTGTAGCCAAA-3′	

IL-8	Forward	5′-CGCCACGTTCTGACCACTTAGC-3′	168
	Reverse	5′-TTGGACACAGTGTTCTTGCCTTGG-3′	

EP_2_	Forward	5′-ATCTCATCGCACTGGCACTGTTG-3′	112
	Reverse	5′-AGCACCAATTCCGTTACCAGCAC-3′	

COX-1	Forward	5′-GCTGATGCTCTTCTCCACGATCTG-3′	119
	Reverse	5′-TAAGGATGAGGCGAGTGGTCTGG3′	

COX-2	Forward	5′-GGTGCCTGGTCTGATGATGTATGC-3′	81
	Reverse	5′-GGATGCTCCTGCTTGAGTATGTCG-3′	

TNF-*α*	Forward	5′- CTTGTTGCCTCCTCTTTTGCTTA-3′	164
	Reverse	5′-CTTTATTTCTCTCAATGACCCGTAG-3′	

IL-6	Forward	5′-AGGAGTGGCTAAGGACCAAGACC-3′	142
	Reverse	5′-CTGACCACAGTGAGGAATGTCCAC-3′	

IL-1*β*	Forward	5′-GCAGAGCACAAGCCTGTCTTCC-3′	198
	Reverse	5′-ACCTGTCTTGGCCGAGGACTAAG-3′	

ER-*β*	Forward	5′-CGGTCTGTCTGAATGTGGTCACTG-3′	183
	Reverse	5′- TTGCCTTGGTCATGGTATCGCTTC-3′	

TGF-*β*_1_	Forward	5′-ATGGTGGACCGCAACAACGC-3′	98
	Reverse	5′- GGCACTGCTTCCCGAATGTCTG-3′	

VEGF-A	Forward	5′-GTGACAAGCCAAGGCGGTGAG-3′	114
	Reverse	5′- CGATGATGGCGTGGTGGTGAC-3′	

## Data Availability

The data used to support the findings of this study are included within the article.
